# Effects of Neuromuscular Electrical Stimulation on Cramp Threshold Frequency and Pain in Adults with Nocturnal Leg Cramps: An EMG-Based Assessment: Randomized Controlled Trial

**DOI:** 10.3390/healthcare14131884

**Published:** 2026-06-28

**Authors:** Su-Jin Kim, Sun-Min Kim, Sang-Hun Jang

**Affiliations:** 1 Department of Health and Medical Sciences, Graduate School of Physical Therapy, Korea National University of Transportation, Jeungpyeong-gun 27909, Republic of Korea; sj960208@naver.com; 2 Department of Physical Therapy, Gimcheon University, Gimcheon-si 39528, Republic of Korea; jjssppaarrkk@hanmail.net; 3 Department of Physical Therapy, College of Health and Life Science, Korea National University of Transportation, Jeungpyeong-gun 27909, Republic of Korea

**Keywords:** cramp threshold frequency, muscle cramp, neuromuscular electrical stimulation, pain, surface electromyography

## Abstract

**Background:** Muscle cramps are sudden, painful, and recurrent contractions that may interfere with physical activity and daily life. Although stretching is commonly used to relieve cramps, the effectiveness of neuromuscular electrical stimulation (NMES) has not been fully established. **Objective:** This study aimed to investigate the effects of NMES on cramp threshold frequency (CTF) and pain intensity in individuals with a history of nocturnal leg cramps without identifiable underlying disorders. **Methods:** Twenty healthy adults with a history of leg cramps were randomly assigned to either an experimental group (*n* = 10) or a control group (*n* = 10). The experimental group received electrical stimulation to both calves using an NMES device, whereas the control group performed lower-extremity static stretching exercises. Both groups participated in an intervention program conducted three times per week for 20 min per session over 4 weeks. CTF was measured using surface electromyography, and muscle cramps were induced using an electrical stimulation device starting at 4 Hz, with the frequency increased by 2 Hz after each stimulation set until cramp onset. Pain intensity was assessed using the Visual Analog Scale (VAS). Within-group and between-group comparisons before and after the intervention were analyzed using paired-samples *t*-tests and independent-samples *t*-tests, respectively, with the level of statistical significance set at *p* < 0.05. **Results:** Both groups showed significant improvements in CTF and pain intensity after the intervention. However, the NMES group demonstrated significantly greater improvement than the stretching group. The increase in CTF was significantly greater in the experimental group than in the control group (9.20 ± 6.75 vs. 1.80 ± 1.75; *t* = 3.357, *p* = 0.007). Likewise, the reduction in VAS score was significantly greater in the experimental group than in the control group (−5.20 ± 0.92 vs. −1.60 ± 0.84; *t* = 9.128, *p* < 0.001). **Conclusions:** NMES may be an effective intervention for increasing cramp threshold frequency and reducing pain in individuals with leg cramps, with greater benefits than lower extremity stretching.

## 1. Introduction

Nocturnal leg cramps (NLC) occur suddenly at night and are characterized by intermittent yet persistent painful involuntary contractions of the calf, hamstring, or foot muscles [1]. Nocturnal leg cramps (NLC) have a prevalence of approximately 33% in the general population aged over 50 years, and the prevalence of sleep-related leg cramps has also been reported to increase with advancing age in Korea [2,3,4]. NLC is considered to be more closely associated with muscle fatigue and neuromuscular dysfunction rather than electrolyte imbalance or other abnormalities [1]. Reported contributing factors include prolonged sitting, prolonged occupational standing, pregnancy, aging, excessive exercise or physical activity, water–electrolyte imbalance, salt deficiency, renal dialysis, and deep vein thrombosis [5]. The key diagnostic features of NLC include (1) severe pain, (2) a duration ranging from a few seconds to up to 10 min, (3) frequent occurrence in the calf or foot, (4) occasional occurrence in the thigh or hamstring muscles, (5) persistent post-cramp pain, (6) sleep disturbance, and (7) psychological distress [2]. In addition to pain itself, individuals who frequently experience cramps have been reported to have poorer sleep quality, more sleep disturbances, and greater daytime sleepiness than those without cramps [6,7]. NLC are difficult to manage due to their unclear pathophysiology, and as there are limited treatment options with proven safety and efficacy, it is important to explore effective therapeutic approaches [7].

Neuromuscular electrical stimulation (NMES) is a method in which electrical stimulation is delivered through electrodes placed over the skin of the target muscle, depolarizing the neuromuscular end plate via motor nerves and inducing skeletal muscle contraction [8,9]. Previous studies have reported that NMES is commonly used to attenuate muscle atrophy and facilitate motor recovery after injury to central motor pathways and that NMES applied to the lower extremities may improve skeletal muscle mass, muscle function, physical performance, and general health status [10,11,12,13]. In addition, NMES has been reported to be effective in increasing blood flow velocity and volume, as well as reducing lower limb edema in individuals with impaired circulation [14,15].

Several clinical studies have investigated the use of NMES to reduce the frequency and severity of leg cramps. Behringer et al. (2014) [16] reported that, in adults with a history of leg cramps, NMES applied to the calf muscles significantly increased the cramp threshold frequency (CTF) of the cramp-inducing muscle after 3 weeks compared with the control group, and this increasing trend was maintained at the 6-week follow-up. CTF is defined as the minimum stimulation frequency required to induce a muscle cramp [17]. Miller et al. (2009) [17] reported that individuals with a history of cramps had significantly lower CTF values than those without such a history. Harmsen et al. (2021) [18] suggested that NMES may help reduce leg cramps in patients with lumbar spinal stenosis or lumbar disk herniation. In addition, Behringer et al. (2018) [19] found that NMES applied to the calf muscles of individuals with spontaneous leg cramps significantly increased CTF and reduced cramp frequency and intensity. They further proposed that NMES may reduce the occurrence of muscle cramps by increasing inhibitory afferent input to alpha motor neurons.

Other interventions for reducing leg cramps, such as stretching and pharmacological therapy, have also been proposed. Hawke et al. (2021) [20] reported low-certainty evidence that calf or hamstring stretching performed for at least 6 weeks may slightly reduce the pain intensity of nocturnal leg cramps compared with a control condition; however, its effect on cramp frequency remained uncertain. Barna et al. (2021) [21] reported that the group receiving magnesium supplementation showed a significant reduction in the frequency of nocturnal leg cramps compared with the placebo group. Although quinine has been suggested to reduce the frequency and intensity of cramps, its efficacy and safety remain inconsistent [22]. In addition, interventions such as compression stockings, vitamin K2 supplementation, stretching, and meditation have been proposed as potential approaches for alleviating lower-extremity cramps [7,23,24].

Although the effects of conventional treatments and supplements appear to be limited, NMES has shown promising results in previous studies [16,18]. However, the current evidence remains insufficient to generalize the preventive effects of NMES on muscle cramps, and objective quantitative indicators such as CTF are still lacking. In recent years, studies have increasingly employed machine learning–based electromyographic signal analysis and data-driven approaches to quantitatively assess and analyze physiological responses such as muscle fatigue and cramping. Within this context, indices such as CTF have attracted attention as potential biomarkers for individualized stimulation parameter optimization [25,26,27,28]. However, these approaches have not yet been sufficiently applied in clinical research, and objective evaluation studies based on NMES and CTF remain limited. Surface electromyography (sEMG) is a representative objective measurement method that enables the quantitative assessment of muscle fatigue through changes in the electrical activity of muscles [29]. Electromyography-based assessment can identify the onset of cramps through muscle activity and enables CTF analysis as an objective measure for evaluating the effects of NMES. Therefore, this study was based on the hypothesis that the application of NMES in individuals with nocturnal leg cramps would increase CTF and reduce pain. The purposes of this study were as follows: first, to investigate the effects of NMES on CTF and pain in individuals with a history of leg cramps; and second, to compare the effects of NMES on CTF and pain with those in individuals who did not receive NMES.

## 2. Materials and Methods

### 2.1. Research Design

This study employed a randomized controlled trial design to investigate the effects of neuromuscular electrical stimulation (NMES) on cramp threshold frequency (CTF) and pain in adults aged 50 years and older with nocturnal leg cramps. Twenty participants who met the inclusion criteria were enrolled. Participants were randomly assigned to either the NMES group or the static stretching group using an opaque-box randomization method. The box contained 20 slips of paper, including 10 labeled “1” and 10 labeled “2.” Participants who selected “1” were allocated to the experimental group, whereas those who selected “2” were allocated to the control group. This study was conducted using a single-blind design, and the participants were blinded to their group allocation.

The study was conducted in the rehabilitation treatment room of C Hospital located in C City between 28 December 2025 and 25 January 2026. Both groups participated in a 4-week intervention program consisting of three sessions per week, 20 min per session, for a total of 12 sessions. Outcome measures were collected before and after the intervention. This study was approved by the Institutional Review Board of Korea National University of Transportation (KNUT-2025-HR-30-45) and was registered with the Clinical Research Information Service (registry number: KCT0011242).

### 2.2. Subjects

The participants in this study were recruited from healthy adults working at C Hospital located in C City who experienced leg cramps and met the study criteria. The inclusion criteria were adults aged 50 years or older who experienced recurrent lower-extremity muscle cramps without evident neurological or vascular disorders and who reported calf muscle cramps during sleep at least twice per week over the previous month. The exclusion criteria were as follows: patients with secondary causes of leg cramps, such as diabetic peripheral neuropathy, peripheral vascular disease, herniated intervertebral disk, or spinal stenosis, and individuals who had been taking medications that could affect muscle cramps, such as calcium channel blockers, for a prolonged period.

### 2.3. Procedure

Following enrollment and randomization, all participants underwent baseline assessment prior to the intervention. The experimental group received NMES, while the control group performed static stretching. Both interventions were administered over 4 weeks, three times per week, for 20 min per session.

**Figure A1:**
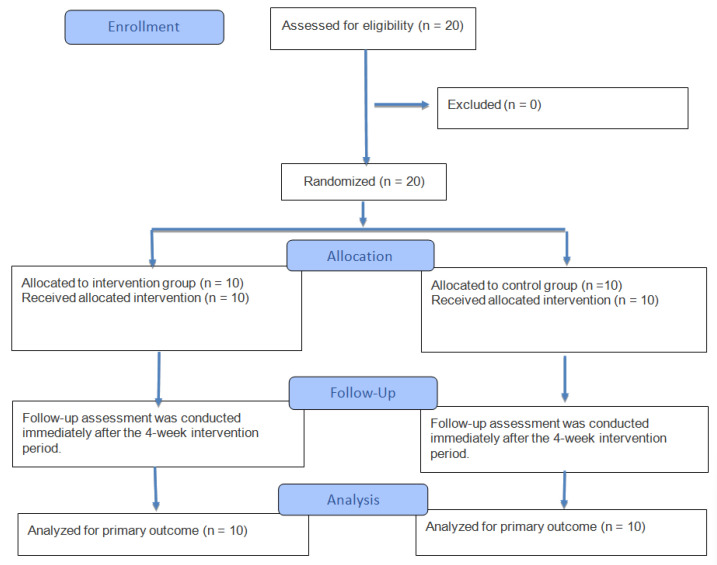
Flow diagram.

After completion of the intervention period, post-intervention assessments were conducted using the same measurement tools applied at baseline. To ensure safety during the assessment process, all evaluations were conducted by a physical therapist with more than 10 years of clinical experience, and one assessment assistant with more than 8 years of clinical experience remained available to provide assistance and supervision when needed. Both the assessor and the assistant were blinded to group allocation. The study flow diagram and CONSORT checklist are presented in Figure A1 and Table A1 of the Appendix A, respectively.

### 2.4. Research Methods

#### 2.4.1. Experimental Group

The experimental group received neuromuscular electrical stimulation (NMES), whereas the control group participated in a leg stretching program aimed at reducing cramps. The NMES device used in the experimental group was a portable stimulator (Double Walking EMS Basic Model, GOS, Gyeongsan-si, Republic of Korea) capable of delivering biphasic rectangular pulses with a pulse width of 500 μs, current intensity of 1–30 mA, and a frequency of up to 80 Hz. To apply the stimulation, water was sprayed onto the silver-thread pads embedded inside the calf strap, the strap was wrapped around the calf, and the NMES unit was attached to the magnet fixed to the outer pocket of the strap. The device allowed individual adjustment of stimulation intensity through built-in control switches.

Participants in the experimental group wore calf straps containing the NMES device on both calves and received electrical stimulation for 20 min. Prior to the intervention, the maximum tolerated stimulation current (mTSC) was determined for each participant. The mTSC was defined as the maximum electrical stimulation intensity tolerable by the participant. The stimulation intensity was initially set at 85% of the mTSC to minimize pain and discomfort [16]. If the participant reported that the stimulation intensity was insufficient, it was increased up to 100% of the mTSC. If pain or discomfort occurred, the stimulation intensity was decreased accordingly.

#### 2.4.2. Control Group

**Figure 1:**
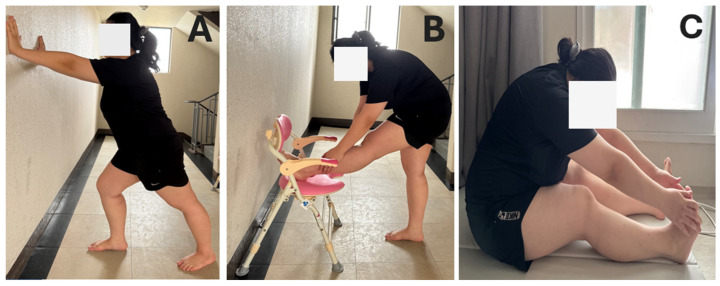
Static stretching program performed by the control group. (**A**) Standing calf and hamstring stretching; (**B**) standing hamstring stretching; (**C**) seated calf and hamstring stretching.

Participants in the control group performed static stretching exercises targeting the calf muscles to reduce the frequency and intensity of spontaneous leg cramps. The stretching program was designed to target the gastrocnemius, soleus, and hamstring muscles and was performed for a total of 20 min based on the protocol described by Hallegraeff et al. [30]. The program consisted of three stretching exercises performed bilaterally, with each stretch held for 30 s followed by a 20 s rest period, for a total of three sets. The exercises included standing calf stretching, standing hamstring stretching, and seated calf and hamstring stretching. For the standing calf stretch, participants placed their hands against a wall, extended one leg backward while keeping the heel on the floor, and bent the front knee while leaning the trunk forward. For the standing hamstring stretch, participants placed one heel on a chair with the knee fully extended and leaned the trunk forward to stretch the hamstring muscles. For the seated hamstring and calf stretch, participants sat on a firm bed or floor with both legs extended, dorsiflexed the ankles, held their feet with their hands, and leaned the trunk forward. The stretching intensity was adjusted according to individual flexibility and was maintained at a level that did not induce pain. The intervention program for the control group is presented in Table 1, Figure 1.

### 2.5. Measurement Items

#### 2.5.1. Surface Electromyography (Ultium EMG, Noraxon, Scottsdale, AZ, USA)

In this study, surface electromyography (sEMG) was used to measure the CTF, defined as the frequency at which a muscle cramp was first induced by electrical stimulation. Surface electromyography is a widely used non-invasive assessment method for evaluating neuromuscular activation in rehabilitation research [31]. The EMG signals were sampled at 2000 Hz and processed using a band-pass filter of 20–500 Hz. For EMG data collection, a 2–3 s resting period before the first stimulation was recorded as the baseline. During the observation window, the 4 s period immediately following the 5 s electrical stimulation was used for analysis. The average root mean square amplitude (aRMSA) was calculated for the observation window and compared with the baseline aRMSA.

At the pre-intervention assessment, the baseline cramp response of all participants was evaluated. A cramp-inducing device (GOS, Gyeongsan-si, Republic of Korea) was used to deliver electrical stimulation, and the CTF, defined as the stimulation frequency at the onset of cramp, was recorded using sEMG. The cramp-inducing device delivered electrical stimulation with a pulse width of 400 μs and a current intensity of 40 mA. The initial stimulation frequency was set at 4 Hz, and stimulation was applied in repeated cycles of 5 s on and 55 s off. After each cycle, the frequency increased automatically by 2 Hz until it reached 40 Hz, at which point the test was terminated. During the assessment, participants were seated in a chair in a relaxed position with the calf muscles fully relaxed. To minimize measurement interference, they were instructed to remain seated without voluntary muscle contraction and to relax the muscles as much as possible [16].

**Figure 2:**
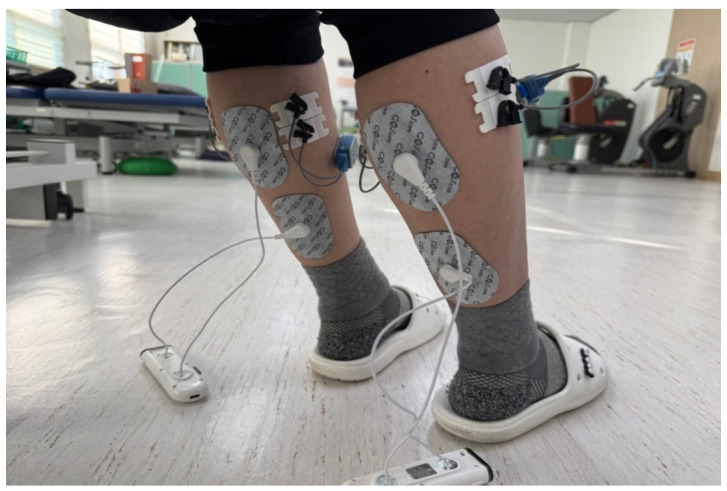
Measurement of cramp threshold frequency using surface electromyography.

The criteria for identifying cramp onset were as follows: (1) when the RMS value exceeded the baseline mean plus two standard deviations; (2) when burst-like EMG activity persisted for more than 1–3 s after the end of the cramp-inducing stimulation; (3) when the participant reported experiencing a cramp; and (4) when sustained involuntary plantar flexion of the ankle was observed immediately after stimulation. When all criteria were met during the assessment, the examiner recorded the corresponding stimulation frequency as the participant’s CTF (Figure 2).

#### 2.5.2. Visual Analog Scale (VAS)

In this study, the VAS was used to record the average intensity of cramp-related pain experienced by the participants. Previous studies have reported that the VAS is more sensitive to subtle changes in pain intensity than the Numeric Rating Scale (NRS) or the Verbal Rating Scale (VRS) [32]. VAS has demonstrated high reliability, with an intraclass correlation coefficient of 0.97 for the total score [33]. In the present study, the VAS was recorded to assess pain severity when a cramp occurred following cramp induction.

### 2.6. Data Analysis

The sample size for this study was determined based on the findings of previous studies [19]. Using G*Power software (version 3.1.9.4), the required sample size was estimated to be 20 participants with a power of 0.80, a significance level of 0.05, and an effect size of 1.325. In this study, data from all 20 participants were analyzed without any dropouts.

All statistical analyses were performed using SPSS version 24.0 (IBM Corp., Armonk, NY, USA). Normality of the data was assessed using the Shapiro–Wilk test. To verify baseline homogeneity of the general characteristics between the two groups, the chi-square test and independent *t*-test were used as appropriate. Within-group pre–post differences were analyzed using the paired *t*-test, and between-group differences in change scores were analyzed using the independent *t*-test. The level of statistical significance was set at *p* < 0.05.

## 3. Results

### 3.1. General Characteristics of the Subjects

In the experimental group, there were 2 males and 8 females, whereas the control group consisted of 3 males and 7 females, with no statistically significant difference in sex distribution between the two groups (χ^2^ = 0.267, *p* = 1.000). The general characteristics of the subjects are presented in Table 2. These findings indicate that the two groups were homogeneous with respect to their general characteristics and baseline outcome measures.

### 3.2. Changes in Cramp Threshold Frequency (CTF)

**Figure 3:**
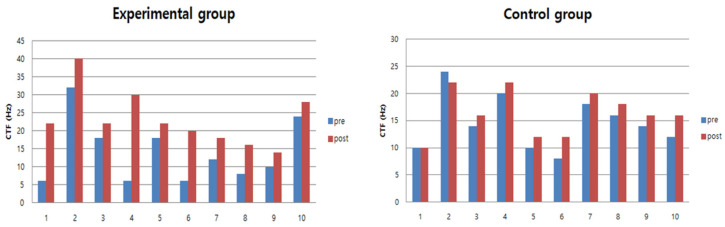
Individual pre–post changes in CTF for each group.

Within-group analysis showed significant pre–post differences in Cramp Threshold Frequency (CTF) in both groups (*p* < 0.05). In the between-group comparison of change scores, the experimental group demonstrated a significantly greater increase in CTF than the control group (9.20 ± 6.75 Hz vs. 1.80 ± 1.75 Hz, *p* < 0.05). These findings indicate that the experimental group showed greater improvement in CTF than the control group (Table 3, Figure 3).

### 3.3. Changes in Visual Analog Scale (VAS)

**Figure 4:**
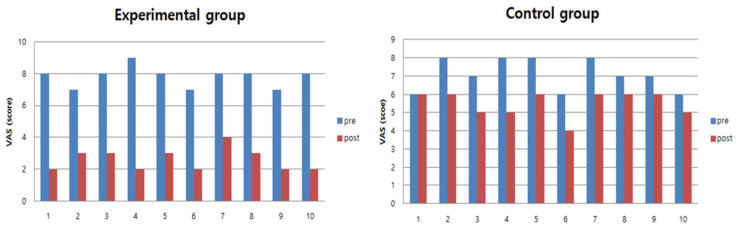
Individual pre–post changes in VAS for each group.

Within-group analysis showed significant pre–post differences in Visual Analog Scale (VAS) scores in both groups (*p* < 0.001). The between-group comparison also showed a statistically significant difference, with the experimental group demonstrating a greater reduction in VAS scores than the control group (−5.20 ± 0.92 points vs. −1.60 ± 0.84 points, *p* < 0.001) (Table 4, Figure 4).

## 4. Discussion

The present randomized controlled trial investigated the effects of neuromuscular electrical stimulation (NMES) on cramp threshold frequency (CTF) and cramp-related pain in adults aged 50 years and older with a history of leg cramps. The principal findings of this study were as follows. First, both the NMES and control groups demonstrated significant improvements in CTF and pain following the intervention. Second, the magnitude of change was significantly greater in the NMES group than in the control group.

In the present study, CTF increased significantly in both groups after the intervention, with a greater improvement observed in the NMES group. CTF is regarded as an objective electrophysiological indicator of muscle cramp susceptibility, and lower CTF values have been reported in individuals with a history of cramps compared with those without such a history [17]. Therefore, an increase in CTF may be interpreted as reflecting reduced neuromuscular excitability and a lower tendency for cramp induction. Notably, in the NMES group of the present study, CTF increased from 14.00 ± 8.84 Hz before the intervention to 23.20 ± 7.67 Hz after the intervention, indicating a substantial reduction in cramp sensitivity.

The present findings are consistent with those of previous studies examining the effects of electrical stimulation on cramp susceptibility. Behringer et al. (2014) [16] reported that NMES applied to cramp-inducing muscles significantly increased CTF in adults with a history of leg cramps. Likewise, Harmsen et al. (2021) [18] found that NMES reduced the frequency of leg cramps and significantly increased CTF, particularly at stimulation intensities of 25% and 85%. These results suggest that repeated neuromuscular stimulation may induce adaptive changes within the neuromuscular system, thereby increasing the threshold for cramp induction. Furthermore, previous research has shown that electrically induced muscle cramp training alone can significantly increase CTF for up to 24 h [34], implying that repetitive stimulation itself may contribute to short-term neuromuscular adaptation. Taken together, these findings suggest that repeated neuromuscular stimulation may be associated with short-term and cumulative changes in cramp sensitivity. Furthermore, it is suggested that muscle fatigue can be assessed using data-driven analyses and machine learning techniques based on electromyographic signals, enabling the individualized adjustment of NMES intensity and frequency.

Several possible explanations may account for the increase in CTF following the NMES intervention. Muscle cramps are generally thought to occur as a result of increased excitatory input from muscle spindles and decreased inhibitory input from Golgi tendon organs, leading to heightened alpha motor neuron excitability. Repeated NMES-induced contractions may influence this imbalance by providing recurrent afferent input and promoting neuromuscular adaptation. Although the neurophysiological mechanisms underlying these effects were not directly examined in the present study, the increase in CTF observed in the NMES group may reflect changes in peripheral and spinal neuromuscular regulation.

In addition to the improvement in CTF, VAS scores decreased significantly in both groups, with the NMES group showing a significantly greater reduction than the control group. This finding indicates that NMES may not only delay the onset of muscle cramps but also attenuate the pain associated with cramp episodes. Several mechanisms may account for this effect. First, NMES-induced muscle contractions may enhance local blood flow and oxygen delivery, thereby reducing ischemic discomfort and the accumulation of metabolites associated with pain. Previous studies have demonstrated that NMES can produce greater muscle blood flow and oxygen consumption than voluntary contraction under comparable workload conditions [35,36,37]. Second, NMES may contribute to pain modulation through activation of inhibitory neural pathways. Repeated stimulation of peripheral nerves and muscle fibers may provide afferent input that suppresses nociceptive transmission at the spinal or supraspinal level. Third, reduced cramp susceptibility itself may contribute to pain relief by decreasing the frequency or severity of cramp episodes.

The present findings regarding pain reduction are also in agreement with previous reports in clinical populations. In a secondary analysis by Talbot et al. (2025) [38], which examined three randomized controlled trials, the NMES group showed a tendency toward reduced pain immediately after treatment. In a study by Zheng et al. (2025) [39], the combined application of NMES and exercise resulted in a significant reduction in pain intensity. Similarly, Sax et al. (2022) [40] reported a statistically significant decrease in VAS scores in the NMES group. Although the clinical populations and pain mechanisms differ from those in the present study, these findings suggest that NMES may have broader analgesic potential across various musculoskeletal and neuromuscular conditions. In the context of the present study, NMES may have reduced pain through a combination of increased local circulation, improved muscle activation, and reduced cramp sensitivity.

It is also noteworthy that the control group showed significant improvements in both CTF and pain. This finding suggests that lower extremity stretching may also be beneficial for individuals with leg cramps. Stretching may reduce passive muscle stiffness, improve flexibility, and influence muscle spindle sensitivity, thereby contributing to a reduction in cramp occurrence and associated pain. Although the magnitude of improvement was greater in the NMES group, it remains unclear whether the difference in change scores reflects a specific effect of electrical stimulation itself, as the comparison intervention was based on passive stretching. But, the present study may provide clinical evidence supporting the use of NMES as an alternative intervention for individuals who have difficulty performing regular stretching programs or as part of an exercise-based management approach.

The present study has several clinical implications. Leg cramps are a common complaint among middle-aged and older adults and may negatively affect sleep quality, physical function, and overall quality of life. Pharmacological treatment may be accompanied by the risk of adverse effects, which could potentially worsen the condition. From this perspective, NMES may represent a practical and clinically relevant intervention because it is non-pharmacological, relatively easy to administer, and applicable in both clinical and home settings. In particular, the use of portable NMES devices may facilitate integration into structured home-based rehabilitation or self-management programs for individuals with recurrent cramps. This may help improve participants’ accessibility to and adherence with the intervention. Accordingly, the findings of this study provide preliminary evidence supporting the potential clinical utility of NMES as part of a conservative treatment strategy for leg cramps.

Several limitations of this study should be acknowledged. First, the sample size was relatively small, which may limit the generalizability of the findings and reduce statistical power. Second, the possibility of a placebo effect cannot be excluded, as participants receiving NMES may have had greater expectations regarding treatment efficacy. Third, factors that may influence cramp occurrence, such as physical activity level, hydration status, sleep quality, medication use, and nutritional status, were not fully controlled. Fourth, the follow-up period was limited to the intervention period, making it difficult to determine whether the observed benefits were maintained over time. Finally, One important limitation is that, because the comparison intervention was based on static stretching, the observed between-group differences in this study cannot be interpreted as reflecting NMES-specific effects alone. Future studies including active contraction-based comparison groups are needed to more clearly distinguish the underlying mechanisms.

Future studies should include larger sample sizes, longer follow-up periods, and more diverse participant populations in order to confirm the long-term effects and generalizability of NMES for the management of leg cramps. Additional research is also needed to identify the optimal stimulation parameters, including intensity, frequency, treatment duration, and total intervention period. Moreover, future investigations should examine whether combining NMES with stretching, strengthening exercise, hydration education, or home-based self-management strategies yields greater therapeutic effects than a single intervention alone. The inclusion of broader outcome measures, such as cramp frequency, sleep disturbance, functional performance, and quality of life, would further strengthen the clinical relevance of subsequent studies. Furthermore, there is a need for individualized NMES application based on the analysis of electromyographic signals reflecting each individual’s neuromuscular response threshold, and additional research is warranted to optimize stimulation intensity and frequency.

## 5. Conclusions

This study aimed to investigate the effects of neuromuscular electrical stimulation (NMES) on cramp threshold frequency (CTF) and pain in individuals with a history of leg cramps by comparing an NMES group with a control group that did static stretching. The results showed that both groups demonstrated statistically significant improvements in CTF and pain, with statistically greater improvement observed in the NMES group. These findings suggest that NMES may be an effective intervention for delaying cramp onset and reducing cramp-related pain in adults with a history of recurrent nocturnal leg cramps.

## Figures and Tables

**Table 1:** Static stretching program of the control group.

Contents	Intervention	Protocol	Session Time (Minute)
Warm up	Postural education and breathing		2
Main stretching exercise	Calf stretching (standing)	30 s × 3 sets 20 s rest	16
	Hamstring stretching (standing)		
	Hamstring and calf stretching (sitting)		
Cool down	Breathing		2

**Table 2:** General characteristics of subjects (*n* = 20).

	Experimental Group (*n* = 10)	Control Group (*n* = 10)	*t*/x^2^ (*p*)
Sex (male/female)	2/8	3/7	0.267 ^b^ (1.000 ^c^)
Age (years)	70.50 ± 3.14 ^a^	70.30 ± 2.87	0.149 (0.883)
Height (cm)	157.50 ± 6.24	158.00 ± 6.46	0.176 (0.862)
Weight (kg)	56.10 ± 8.93	56.70 ± 7.92	0.159 (0.875)
CTF	14.00 ± 8.84	14.60 ± 4.99	0.187 (0.854)
VAS	7.80 ± 0.63	7.10 ± 0.88	2.049 (0.055)

Note. ^a^ Mean ± SD, ^b^ Chi-square test, and ^c^ independent *t*-test. experimental group, neuromuscular electrical stimulation group; control group, lower extremity stretching group; CTF, Cramp Threshold Frequency; VAS, Visual Analog Scale.

**Table 3:** The comparison of CTF values between the experimental group and the control group.

		Experimental Group (*n* = 10)	Control Group (*n* = 10)	*t*	*p*	95% CI
CTF	Pre	14.00 ± 8.84 ^a^	14.60 ± 4.99			
	Post	23.20 ± 7.67	16.40 ± 4.20			
	Post–Pre	9.20 ± 6.75	1.80 ± 1.75	3.357	0.007 *	(2.77, 12.03)
	*t* (*p*)	4.313 (0.002 *)	3.250 (0.010 *)			

Note. ^a^ Mean ± SD; CTF, cramp threshold frequency; CI, confidence interval; and * *p* < 0.05. Experimental group, neuromuscular electrical stimulation group; control group, lower extremity stretching group.

**Table 4:** The comparison of VAS values between the experimental group and the control group.

		Experimental Group (*n* = 10)	Control Group (*n* = 10)	*t*	*p*	95% CI
VAS	Pre	7.80 ± 0.63 ^a^	7.10 ± 0.88			
	Post	2.60 ± 0.70	5.50 ± 0.71			
	Post–Pre	5.20 ± 0.92	1.60 ± 0.84	9.128	0.000 *	(−4.42, −2.78)
	*t* (*p*)	17.894 (0.000 *)	6.000 (0.000 *)			

Note. ^a^ Mean ± SD, VAS, Visual Analog Scale, CI, Confidence Interval, and * *p* < 0.05. experimental group, neuromuscular electrical stimulation group; control group, lower extremity stretching group.

## Data Availability

The original contributions presented in this study are included in the article. Further inquiries can be directed to the corresponding author.

## Appendix A

**Table A1:** CONSORT checklist.

Section/Topic	No	CONSORT 2025 Checklist Item Description	Reported on Page No.
Title and abstract			
Title and structured abstract	1a	Identification as a randomized trial	1
	1b	Structured summary of the trial design, methods, results, and conclusions	1
Open science			
Trial registration	2	Name of trial registry, identifying number (with URL) and date of registration	1
Protocol and statistical analysis plan	3	Where the trial protocol and statistical analysis plan can be accessed	1
Data sharing	4	Where and how the individual de-identified participant data (including data dictionary), statistical code and any other materials can be accessed	11
Funding and conflicts of interest	5a	Sources of funding and other support (e.g., supply of drugs), and role of funders in the design, conduct, analysis and reporting of the trial	11 (funding X)
	5b	Financial and other conflicts of interest of the manuscript authors	11
Introduction			
Background and rationale	6	Scientific background and rationale	2
Objectives	7	Specific objectives related to benefits and harms	2
Methods			
Patient and public involvement	8	Details of patient or public involvement in the design, conduct and reporting of the trial	3
Trial design	9	Description of trial design including type of trial (e.g., parallel group, crossover), allocation ratio, and framework (e.g., superiority, equivalence, non-inferiority, exploratory)	3
Changes to trial protocol	10	Important changes to the trial after it commenced including any outcomes or analyses that were not prespecified, with reason	3
Trial setting	11	Settings (e.g., community, hospital) and locations (e.g., countries, sites) where the trial was conducted	3
Eligibility criteria	12a	Eligibility criteria for participants	3
	12b	If applicable, eligibility criteria for sites and for individuals delivering the interventions (e.g., surgeons, physiotherapists)	4
Intervention and comparator	13	Intervention and comparator with sufficient details to allow replication. If relevant, where additional materials describing the intervention and comparator (e.g., intervention manual) can be accessed	4, 5
Outcomes	14	Prespecified primary and secondary outcomes, including the specific measurement variable (e.g., systolic blood pressure), analysis metric (e.g., change from baseline, final value, time to event), method of aggregation (e.g., median, proportion), and time point for each outcome	5–7
Harms	15	How harms were defined and assessed (e.g., systematically, non-systematically)	
Sample size	16a	How sample size was determined, including all assumptions supporting the sample size calculation	6, 7
	16b	Explanation of any interim analyses and stopping guidelines	
Randomization:			
Sequence generation	17a	Who generated the random allocation sequence and the method used	3
	17b	Type of randomization and details of any restriction (e.g., stratification, blocking and block size)	3
Allocation concealment mechanism	18	Mechanism used to implement the random allocation sequence (e.g., central computer/telephone; sequentially numbered, opaque, sealed containers), describing any steps to conceal the sequence until interventions were assigned	3
Implementation	19	Whether the personnel who enrolled and those who assigned participants to the interventions had access to the random allocation sequence	3
Blinding	20a	Who was blinded after assignment to interventions (e.g., participants, care providers, outcome assessors, data analysts)	3
	20b	If blinded, how blinding was achieved and description of the similarity of interventions	3
Statistical methods	21a	Statistical methods used to compare groups for primary and secondary outcomes, including harms	6, 7
	21b	Definition of who is included in each analysis (e.g., all randomized participants), and in which group	6, 7
	21c	How missing data were handled in the analysis	
	21d	Methods for any additional analyses (e.g., subgroup and sensitivity analyses), distinguishing prespecified from post hoc	6, 7
Results			
Participant flow, including flow diagram	22a	For each group, the numbers of participants who were randomly assigned, received intended intervention, and were analyzed for the primary outcome	7, 8
	22b	For each group, losses and exclusions after randomization, together with reasons	
Recruitment	23a	Dates defining the periods of recruitment and follow-up for outcomes of benefits and harms	3
	23b	If relevant, why the trial ended or was stopped	
Intervention and comparator delivery	24a	Intervention and comparator as they were actually administered (e.g., where appropriate, who delivered the intervention/comparator, how participants adhered, whether they were delivered as intended (fidelity))	4
	24b	Concomitant care received during the trial for each group	
Baseline data	25	A table showing baseline demographic and clinical characteristics for each group	7
Numbers analyzed, outcomes and estimation	26	For each primary and secondary outcome, by group: the number of participants included in the analysis the number of participants with available data at the outcome time point result for each group, and the estimated effect size and its precision (such as 95% confidence interval) for binary outcomes, presentation of both absolute and relative effect size	7, 8
Harms	27	All harms or unintended events in each group	
Ancillary analyses	28	Any other analyses performed, including subgroup and sensitivity analyses, distinguishing pre-specified from post hoc	
Discussion			
Interpretation	29	Interpretation consistent with results, balancing benefits and harms, and considering other relevant evidence	8–11
Limitations	30	Trial limitations, addressing sources of potential bias, imprecision, generalizability, and, if relevant, multiplicity of analyses	10
